# Patient and public attitudes towards informed consent models and levels of awareness of Electronic Health Records in the UK

**DOI:** 10.1016/j.ijmedinf.2015.01.008

**Published:** 2015-04

**Authors:** Fiona Riordan, Chrysanthi Papoutsi, Julie E. Reed, Cicely Marston, Derek Bell, Azeem Majeed

**Affiliations:** a NIHR CLAHRC for Northwest London, Imperial College London, Chelsea & Westminster Hospital NHS Foundation Trust, London, United Kingdom; b Department of Social and Environmental Health Research, London School of Hygiene and Tropical Medicine, London, United Kingdom; c Department of Primary Care & Public Health, Imperial College London, London, United Kingdom

**Keywords:** Electronic health records, Informed consent, Patient attitudes, Information governance, Policy

## Abstract

**Background:**

The development of Electronic Health Records (EHRs) forms an integral part of the information strategy for the National Health Service (NHS) in the UK, with the aim of facilitating health information exchange for patient care and secondary use, including research and healthcare planning. Implementing EHR systems requires an understanding of patient expectations for consent mechanisms and consideration of public awareness towards information sharing as might be made possible through integrated EHRs across primary and secondary health providers.

**Objectives:**

To explore levels of public awareness about EHRs and to examine attitudes towards different consent models with respect to sharing identifiable and de-identified records for healthcare provision, research and planning.

**Methods:**

A cross-sectional questionnaire survey was administered to adult patients and members of the public in primary and secondary care clinics in West London, UK in 2011. In total, 5331 individuals participated in the survey, and 3157 were included in the final analysis.

**Results:**

The majority (91%) of respondents expected to be explicitly asked for consent for their identifiable records to be accessed for health provision, research or planning. Half the respondents (49%) did not expect to be asked for consent before their de-identified records were accessed. Compared with White British respondents, those from all other ethnic groups were more likely to anticipate their permission would be obtained before their de-identified records were used. Of the study population, 59% reported already being aware of EHRs before the survey. Older respondents and individuals with complex patterns of interaction with healthcare services were more likely to report prior awareness of EHRs. Individuals self-identifying as belonging to ethnic groups other than White British, and those with lower educational qualifications were less likely to report being aware of EHRs than White British respondents and respondents with degree-level education, respectively. Those who reported being aware of EHRs were less likely to say they expected explicit consent to be sought before use of their de-identified record.

**Conclusions:**

A large number of patients remain unaware of EHRs, while preference for implicit consent is stronger among those who report previous awareness. Differences in awareness levels and consent expectations between groups with different socio-demographic characteristics suggest that public education and information campaigns should target specific groups to increase public awareness and ensure meaningful informed consent mechanisms.

## Highlights

• Most participants would prefer to opt-in before their *identifiable* records are used.
• Half of participants would share their *de-identified* records under implicit consent.
• A low awareness of EHRs persists among participants.
• Participants who were aware of EHRs were more willing to share de-identified data.
• Awareness and consent expectations vary by socio-demographic characteristics.

## 1 Introduction

The potential to reuse health information collected in clinical settings for planning and research purposes has been a major driver for the diffusion and integration of Electronic Health Records (EHRs) in the English National Health Service (NHS) [1–3]. UK government initiatives, such as the data extraction service from primary care (‘care.data’) [4] and the Clinical Practice Research Datalink (CPRD) [5] are underway to maximise the usefulness of health information within existing EHRs. A number of benefits can potentially be realised through the use of integrated records, including improved health outcomes, enhanced quality of clinical research and effectiveness of medical care services [6,7]. However, the effort to increase and systematise health information sharing for secondary purposes has raised a debate among health professionals, patient groups and privacy advocates. In particular, reactions have focused on whether patients and the public are aware of the proposed changes and whether appropriate consent mechanisms are in place [8–12].

Informed consent is important in navigating the balance between the potential benefit and harm from information sharing for secondary purposes, while offering patients control over their personal data [13–16]. Yet, there is little agreement about what constitutes meaningful and informed consent in the case of networked, integrated EHRs, and what options should be available to individuals who wish to revoke any permission to use this data [17–19]. The recent UK Information Governance review led by Dame Fiona Caldicott recommended that de-identified data ‘with a low residual risk of re-identification’ should be shared under an implied consent model, where patient permission to use these data is regarded as implicit or assumed [20]. The same report concluded that identifiable data should be treated as more complex and only shared on a ‘need to know’ basis under explicit consent-seeking mechanisms, where data sharing is not assumed to be automatically permitted [20].

Previous research on patient views about consent mechanisms has identified three important factors that influence individuals’ decisions to allow their data to be used: (1) the perceived sensitivity of the data; (2) the nature of patient interaction with, and trust, in the data recipient; and (3) the extent to which individuals feel informed about how their data will be used [21–26]. Despite the range of secondary purposes for which health data may be shared, work carried out to date has generally focused on public attitudes to data sharing for research purposes only [15,16,21,25–35], with a smaller number of studies examining the use of data for research along with healthcare planning and policy [22–24,36], or for any purpose other than direct patient care [37,38]. As such, research on public attitudes about integrated EHRs used simultaneously for multiple purposes, has been relatively limited, and has not incorporated the potential for wider sharing afforded by EHRs, and the public response in terms of their preferences. Furthermore, most studies which have examined consent preferences in terms of patient characteristics, have been conducted outside the UK [33,35,39–41], or have been limited to one or two sampling sites [23,33,42]. The relationship between consent and socio-demographic factors has also been variable across studies in terms of the magnitude and direction of effect [43,44].

To achieve meaningful and informed consent processes, it is imperative that individuals understand what health information sharing entails [13,14,17,19,45]. However, the published research suggests that people are poorly informed about the uses of their health records and the reasons records may be accessed [25,26]. The UK is no exception: patient awareness of the details of health information sharing in the NHS is low, with a number of studies reporting a lack of meaningful patient engagement, despite information campaigns about the Summary Care Records programme and other initiatives [15,21,22,32,43,46,47].

Despite general lack of awareness, individuals who do report familiarity with EHRs and those who understand how their data would be used are more likely to support EHR use in general and be willing to share their data for research [15,39,43,48]. However, previous work has not made explicit the link between awareness of EHRs and support for the different consent models intended to govern health data sharing. More work is required to identify levels of patient and public awareness about the use of longitudinal, fully comprehensive EHRs and to examine how these relate to expectations of implicit and explicit consent models.

In this paper we examine levels of support for implied and explicit consent models with respect to sharing identifiable and de-identified health information for multiple purposes, including healthcare provision, research and planning. Furthermore, we explore public awareness about detailed EHRs used simultaneously for these purposes. Specifically, we address the following questions:

1. What is the level of patient and public support for explicit (where patient permission is actively sought) and implicit (where permission is assumed) consent models before the use of identifiable and de-identified EHRs?
2. What is the level of public awareness towards EHRs?
3. How are socio-demographic variables, healthcare interaction patterns and computer expertise associated with support for different consent models, and with awareness of EHRs?
4. What is the relationship between awareness of EHRs and support for different consent models for wider sharing of health information?

## 2 Methods

We conducted a cross-sectional, self-completed questionnaire survey using a stratified cluster random sampling design in an area of West London (UK) over a period of 6 weeks from 1st August 2011. We recruited participants at eight outpatient waiting areas of a teaching hospital, and the waiting rooms of eight general practice (GP) health centres within the hospital catchment area. Eligible participants were over 18 years of age, able to understand the survey information, and had not previously filled out the survey. The study was approved by the London Dulwich Research Ethics Committee (Ref. No. 10/H0808/96).

The 31-item questionnaire explored several aspects of patient and public attitudes towards EHRs (full details available in [49]). The analysis detailed here focuses on three key questions:

1. If your record was part of a national electronic records system, would you expect to be asked before your records were accessed for any reason, if your name and address were present? (‘Yes’; ‘No’).
2. If your record was part of a national electronic records system, would you expect to be asked before your records were accessed for any reason, if your name and address were removed? (‘Yes’; ‘No’).
3. Have you ever heard anything about ‘electronic health records’? (‘Yes’; ‘No’). People who responded positively were then asked about the source of information (media, internet, NHS, word of mouth and/or another source).

The questions on consent referred to an individual's electronic health record, which was described in the survey as ‘computer records with complete and detailed health information’. Although the removal of name and address might not ensure anonymity, we used these measures here as being most relevant to conceptions of personal identity, to denote the difference between identifiable and de-identified records. By mentioning that these identifiers would be removed, we also refer to situations were these would not be visible by, for example, researchers performing database queries.

Additional questions captured socio-demographic characteristics and other personal information (birth year, sex, ethnicity, highest educational qualification attained, confidence with computers), as well as patterns of personal healthcare use (types of health services accessed in the six months before the survey).

Only respondents who provided complete data across all independent and dependent variables of interest were included in the final sample (*N* = 3157). We examined the frequencies of the missing values and used Pearson's Chi-squared test to establish whether differences existed between the analysis sample and the missing sample in the socio-demographic factors, and the distribution of responses on the three questions of interest. Using logistic regression we determined whether certain factors were associated with an individual's inclusion in the final analysis sample.

Descriptive statistics were used to summarise the characteristics of respondents included in the survey sample, and to examine the distribution of responses on the three questions of interest. We examined bivariate associations between the three outcome variables, and between each of the outcome questions and the independent variables, using Pearson's Chi-squared test. Using a multivariate regression model, the associations between each of two outcome questions (consent for de-identified EHRs use and awareness of EHRs) and the socio-demographics and healthcare use were analysed, adjusting the regression analysis for potential clustering at each of the recruitment sites. As a theoretically important consideration, prior awareness of EHRs was also included as an independent variable in the multivariate model for consent preferences. Each regression model was assessed using Hosmer-Lemeshow's goodness of fit test, specifying a grouping of 10. We reported all results at the 95% significance level, and performed the analysis using Stata IC version 11. Full details of the study protocol and the original survey questionnaire have been published elsewhere [49].

## 3 Results

### 3.1 Study population

In total, 5331 individuals participated in the survey: an 85.5% response rate overall for the full questionnaire. Of these, 3157 (59%) provided complete data for the variables analysed here and were included in the final analysis for this paper. The profile of the study sample is shown in Table 1. The sample was 60% female, 55% White British, and had a mean age of 43 (SD = 16). 54% had at least degree-level qualifications, and 78% reported they were confident or very confident computer users. The majority of respondents (68%) had accessed fewer than three types of health services in the six months before the survey. The types of health services specified in the survey included the following: hospital-based services (emergency, planned day visits, planned overnight visits, or planned outpatients visits), NHS Direct, general practice surgeries, GP out-of-hours services, walk-in or out-of-hours clinics.

### 3.2 Levels of support for implicit and explicit consent options

**Fig. 1:**
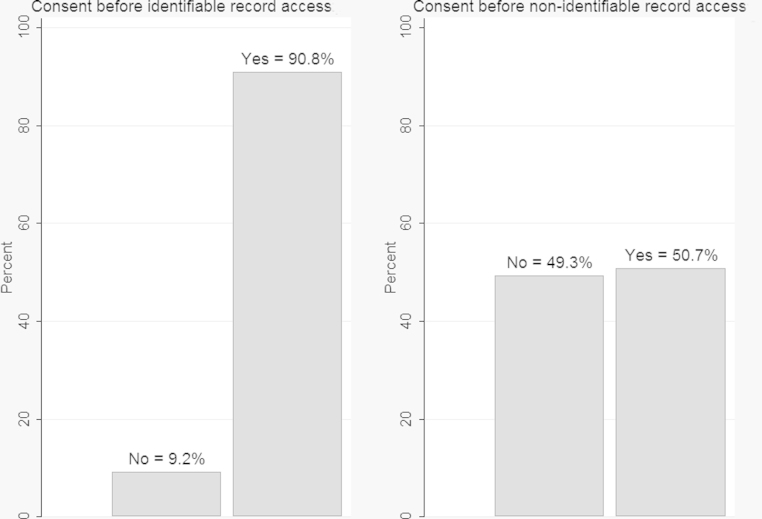
Consent expected before the use of identifiable and de-identified EHRs (*N* = 3157).

Respondents’ levels of support for implicit and explicit consent models for access to identifiable and de-identified records are shown in Fig. 1. Most reported that they would expect to be explicitly asked for consent before their identifiable record was accessed (91%). However, half (49%), reported that they would not expect to be asked for permission before their de-identified record was accessed.

### 3.3 Levels of public awareness

**Fig. 2:**
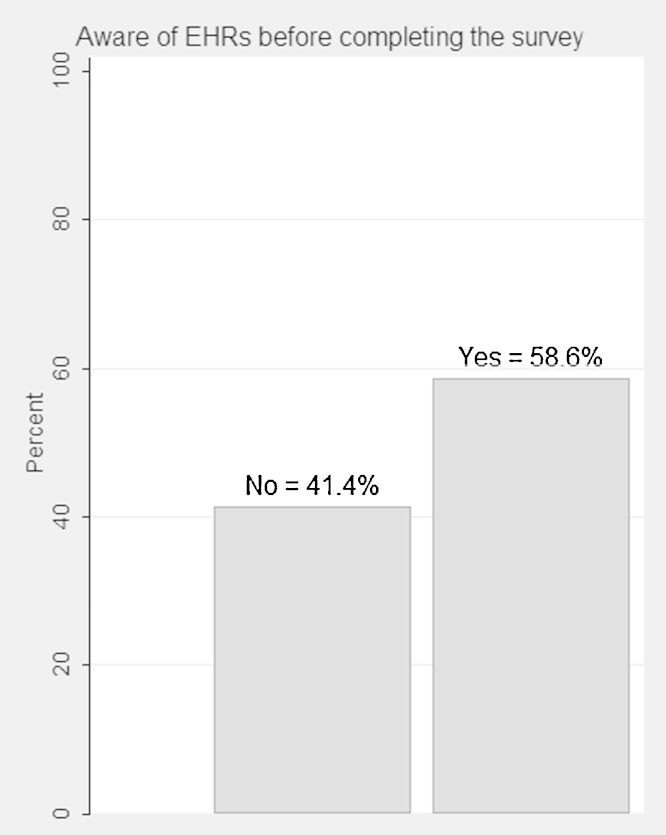
Public awareness of EHRs: proportions of people who had ever heard about EHRs and those who reported not being aware of the concept (*N* = 3157).

A moderately high proportion of respondents reported prior awareness of EHRs (59%), while the remainder had not previously heard about the concept of detailed, longitudinal electronic records used for care, research and policy (Fig. 2).

**Fig. 3:**
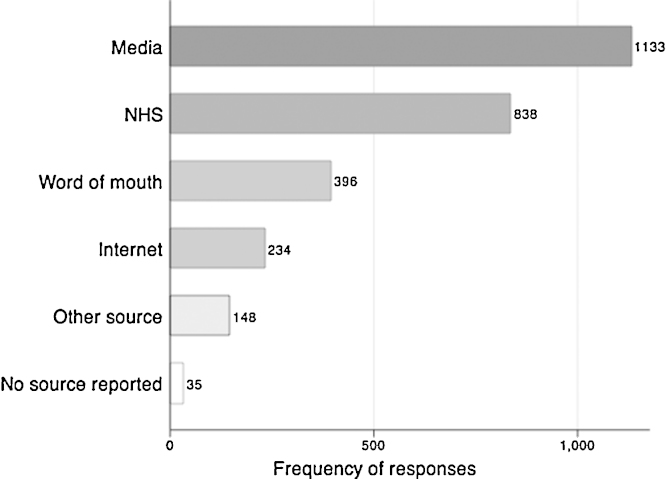
Sources of information on EHRs as reported among respondents who reported ever having heard of EHRs (*N* = 1851 (of Total *N* = 3157)). ^a,b^, ^a^ The question asked was: If yes, to previously hearing about ‘electronic health records’, where have you heard about electronic health records before? (‘The media’; ‘The internet’; ‘The NHS’; ‘Word of mouth’; ‘Another source’) ^b^ The frequencies do not sum to 1851 as more than one option could be selected by respondents.

Respondents who reported having heard of EHRs, most frequently cited the media (i.e. newspaper, radio, television) and the NHS as sources of information (Fig. 3).

### 3.4 Associations between support for consent options for de-identified EHRs, and characteristics of the sample population

Associations between consent options for access to de-identified EHRs and socio-demographic characteristics, computer confidence, healthcare use patterns and prior awareness of EHRs are shown in Table 2. After adjusting for other variables, men were less likely than women to expect consent to be sought before their de-identified records were accessed (OR = 0.66, *P* < 0.001).

People who self-identified as White Non-British (OR = 1.46, *P* < 0.001), British Black (OR = 1.71, *P* < 0.001), Asian British (OR = 1.79, *P* < 0.001) or as belonging to other ethnic groups (OR = 1.66, *P* < 0.001), were all more likely to expect to be explicitly asked for their permission before de-identified record access, compared with White British respondents.

Individuals who reported they were not confident (OR = 1.65, *P* < 0.001) or were only fairly confident (OR = 1.38, *P* < 0.001) in their computer skills, were more likely to expect to give explicit consent for de-identified health records access, compared with those who were very confident. Those with GCSE (OR = 1.61, *P* < 0.05) or A-levels only (OR = 1.47, *P* < 0.001) were more likely to say they thought they should give explicit consent before their de-identified records were shared, than those with a higher degree. Those aware of EHRs were more likely to accept the implicit consent model for use of their de-identified record than those who were unaware of EHRs (OR = 0.77, *P* < 0.001).

The model for de-identified records was judged to be a relatively good fit (Hosmer Lemeshow test statistic *P* = 0.8). A logistic regression model of the association between consent expectations for identifiable records and participant characteristics did not return any statistically significant results, probably because most respondents expected their permission to be sought before any access to their records and the remaining group was small. There was also no association between awareness of EHRs and consent options with respect to identifiable records.

### 3.5 Associations between previous awareness of EHRs, and characteristics of the sample population

Adjusted associations between previous awareness of EHRs and socio-demographic characteristics, confidence with using computers and patterns of healthcare use are illustrated in Table 3. Individuals over 35 years old were more likely to report having previously heard of EHRs, compared with those aged 25–34. Those stating that they had accessed three or more different types of health services in the previous six months, were more likely to have heard of EHRs (OR = 1.27, *P* < 0.05), than those accessing fewer services (0–2 types).

Those self-identifying as White Non-British (OR = 0.43, *P* < 0.001), Black British (OR = 0.64, *P* < 0.05), and Asian British (OR = 0.63, *P* < 0.001) were less likely to have previously heard of EHRs, compared with respondents self-identifying as White British. Individuals with fewer educational qualifications were also less likely to say they had heard about EHRs, compared with respondents with a higher degree (OR = 0.68 to 0.23, *P* < 0.001 in all cases). A similar pattern was observed for those less confident in their computer skills. As reported confidence in computer skills declined, people were less likely to say they had heard about EHRs (OR = 0.79 to 0.26, *P* < 0.001 in all cases), in comparison with those who were very confident.

After inclusion of all relevant independent variables, the model was judged to be a good fit (Hosmer Lemeshow test statistic *P* = 0.1).

### 3.6 Missing data

Only respondents who completed all questions for the variables analysed in this paper were included in the sample. The missing data analysis (Table 4) showed that those who self-identified as Black British were less likely to be included in the analysis sample (OR = 0.64, *P* < 0.05) than White British participants. Similarly, those aged over 45 were less likely to be included (OR declining from 0.66 to 0.27 across age categories, *P* < 0.001 in all cases), compared with those aged 25–34 years.

Respondents with educational qualifications lower than degree level (OR = 0.30 to 0.85, *P* < 0.001 in all cases), and respondents less confident with computers (OR = 0.32 to 0.47, *P* < 0.001 in all cases), were less likely to be included in the sample, compared with those with higher degrees and those very confident with computers, respectively. Participants included in the sample were more likely to have accessed three or more types of healthcare services in the six months before the survey (OR = 1.35, *P* < 0.001).

Respondents who expected to be asked for explicit consent before their identifiable record was accessed were more likely to be included in the complete sample (OR = 1.29, *P* < 0.05). However, those who expected to be asked for explicit consent before access to their de-identified record were less likely to be included in the complete sample (OR = 0.59, *P* < 0.001). In addition, individuals unaware of EHRs before the survey were less likely to be included in the complete sample (OR = 0.62, *P* < 0.001), than those who reported having heard about EHRs.

## 4 Discussion

### 4.1 Principal findings

In relation to public and patient views on consent options for data sharing, this study illustrates that the majority of respondents (91%) would expect to be explicitly asked for consent before their identifiable EHR is accessed, regardless of the reason for access—including for use by healthcare professionals. When sharing de-identified records (name and address removed), fewer participants (51%) said they would expect explicit consent to be sought before data sharing for care, research and healthcare planning. Socio-demographic factors and personal characteristics were further associated with consent preferences. Respondents who identified themselves as belonging to an ethnic group other than ‘White British’, or who were less confident with computers, and those with lower educational qualifications were more likely to expect to be asked for explicit consent before their de-identified records were accessed.

In terms of awareness of EHRs, many participants reported having heard of EHRs before taking part in the survey. However, a sizable minority (41%) reported not being aware of EHRs. Older respondents were more likely to have heard of integrated records. Individuals who identified themselves as belonging to non-White British ethnic groups were less likely to report being aware of EHRs. Those with lower educational qualifications and those reporting less confidence in using computers were also less likely to report having heard of EHRs. Participants who had interacted more with health services seemed to be more exposed to information about EHRs, and the NHS was the second most frequent source of information. Awareness of EHRs was associated with a greater likelihood of reporting acceptance of implicit consent as a model to govern de-identified health information sharing.

As participants reported not being aware of EHRs before the survey, some could have been formulating their views at the time of completing the questionnaire. Responses might have also depended upon how confident people were that the removal of name and address as personal identifiers would be effective enough to protect their privacy. Similar issues around the articulation of patient and public attitudes on EHRs have been commonly discussed in previous studies [22,28], and should be taken in account when interpreting the data presented here, and considered in future research.

### 4.2 Previous studies

This study extends the literature by more closely examining consent preferences in terms of personal characteristics, and by considering the public response to longitudinal, comprehensive EHRs used simultaneously for multiple purposes, rather than just for research or for health services planning. As such, it provides a more robust understanding of how different socio-demographic groups view available consent options in the UK. When considering multiple uses of EHRs, our study indicates that people with different educational qualifications and those belonging to different ethnic groups did not have the same expectations about being explicitly asked for consent before their de-identified records were shared. This extends previous research on the role of education and ethnicity in consenting to record linkage in other countries (e.g. Taiwan [40] and Australia [35]), as well as with regard to consent preferences for linking administrative health data of restricted scope with the British Household Panel Survey in the UK [50].

This work is one of the few studies to directly examine levels of EHR awareness. Although previous research has discussed lack of meaningful public engagement with NHS information campaigns on health records (e.g. [46]), awareness of record sharing for multiple purposes has not been adequately assessed at a similar scale in the UK. A comparable study has been carried out in Canada and shows consistent findings in that older, more educated individuals, with increased healthcare service utilisation, are more likely to be aware of EHRs [39]. The work presented here, however, extends the findings of the Canadian study by showing that individuals self-reporting as being from ethnic groups other than White British or being less confident computer users are less likely to have heard of EHRs. Other research has suggested that participants who are better informed about how their health data is utilised may be more likely to consent to its use [15,43], a premise supported in this study by the association we found between awareness of EHRs and what people expect in terms of consent options for electronic data sharing.

This study also supports previous research suggesting that the majority of individuals would rather be asked for explicit consent, not only before their identifiable health records are accessed [15,21,24,30,43], but also before access to their de-identified records [25,28,37].

### 4.3 Strengths and limitations

This study has the advantage of being carried out using a large sample population, which can be considered fairly representative of West London, when compared with local authority and patient population statistics. Our sample, however, reflects more ethnic diversity than present in the total of the UK population, which allowed us to capture different viewpoints, but should also be taken in account when attempting to generalise the findings presented here. The response rate (86%) for this survey was considerably higher than previous studies examining public attitudes to information sharing [21,23,39].

This is one of the few UK studies of this scale to link patient characteristics to preferences for consent options when sharing de-identified EHRs. It is also the first study to determine what differences might exist between groups who have, and have not heard, of EHRs in the UK, providing useful insights for the development of effective information campaigns on data sharing.

Although we adjusted for key, potentially confounding variables in the analysis, other possible confounding factors including socio-economic measures, such as income level or occupation, may have had an unmeasured further impact. The missing data analysis also demonstrates that respondents unaware of EHRs, those belonging to older age groups, individuals with fewer educational qualifications, those less confident in their computer skills and those self-reporting as Black British, were more likely to be excluded from the analysis sample. This could mean that the views of these groups need to be explored in more detail in future research.

This survey was designed to approach the topic of EHRs from a broad perspective, rather than focusing on specific technological systems. In doing this, we aimed to interrogate patient and public views on processes and principles of data sharing, instead of looking at existing structures which, at time of the survey, were constantly fluctuating (e.g. National Programme for IT). Although this approach generated an understanding of consent expectations that would be applicable to different contexts, differences in opinion depending on particular types of information or sharing arrangements might not have been captured adequately. For example, the high-level survey question on consent does not inform participants about any particular confidentiality safeguards in place or the purpose for which their record would be accessed. Participants were also invited to consider different consent options without receiving any information on potential benefits from record access, which may have elicited different responses (for example see [51]). Research on patient and public views about the specificities of different technological processes, taking in account the way these are supported by situated governance protocols and ethical standards, might provide a more detailed view of how people expect to be asked for consent in different cases.

### 4.4 Implications for research and policy

Current policy initiatives need to take into account that there could be a discrepancy between data sharing in practice and what individuals expect in terms of consent options: a large number of NHS patients are likely to expect to be asked for their explicit consent even to share de-identified records. The study presented here points to the difficulties of achieving wide consensus with respect to sharing de-identified data, particularly when seeking blanket consent for multiple secondary purposes. While there is an emerging focus on dynamic consent models which offer more customisable revocation options and have the potential to become better tailored to patient needs [18], these are not currently fully operational in the NHS. As such, there is still a need to determine how best to achieve wider sharing of patient data through integrated EHR systems in the UK, while recognising patient concerns and preferences. While data sharing plans have changed since the administration of this survey in the UK, high-level public and patient views reported here are still relevant to inform and guide policy as it continues to develop. Core lessons on socio-demographic characteristics in relation to consent and also awareness of EHRs, along with the relationship between the two, are still relevant to inform UK policy as it continues to evolve, and should be taken in account.

Previous research has indicated that further public discussion about EHRs is needed to develop a ‘mutual understanding’ of proposed data sharing arrangements between patients and professionals [52]. The findings reported here suggest that many individuals in the UK remain unaware of EHRs and data sharing plans. Information campaigns may need to be tailored for specific population groups where information about EHRs may otherwise be absent. Our study suggests for instance, specific need for more information among individuals with lower (less than degree level) educational qualifications and individuals who identify themselves as belonging to an ethnic group other than White British. These target groups might be of particular interest to the Department of Health for the information campaign planned about the new GP data extraction service [53]. Apart from general awareness raising on health information sharing plans, it remains important to increase education about governance arrangements, supervision processes and relevant ethical standards.

This study further highlights a relationship between awareness of EHRs and greater contact with health services, which suggests healthcare providers are uniquely positioned to engage patients in productive dialogue and deliberation about their hopes and concerns around wider information sharing for care, research and policy purposes. In this respect, the results of this study support the recommendation made by a recent Information Governance Review, that individuals working in health and social care have a key role in creating an informed public [20].

## 5 Conclusions

This study indicates that most members of the public expect to be asked for explicit consent before their *identifiable* data stored within integrated EHRs is shared for health provision, research and policy. Even for *de-identified* health records, however, half of the respondents expect their explicit consent to be sought. Because those aware of EHRs were more willing to share de-identified data without their express consent, public education may be a way to increase support for implicit consent to share information for care, research and policy. Awareness of EHRs varies between different groups, and NHS information campaigns must ensure all members of the public are adequately informed about the uses of their personal health data.

**Summary points**

What was already known on the topic?

• There are many benefits and risks to integrated EHRs allowing wider sharing of medical information for health provision, research and policy.
• Implementing EHR systems in the UK requires an understanding of patient expectations for consent mechanisms and consideration of public awareness.

What this study added to our knowledge?

• The vast majority of participants expected to be asked for explicit consent (opt-in) before their *identifiable* records were shared for health provision, research and policy.
• Half of the respondents expected that their *de-identified* health records may be used under their implicit consent for health provision, research and policy.
• There is relatively low awareness of EHRs among participants, while those aware of EHRs were more willing to share de-identified data without their express consent.
• There are differences in awareness levels and consent expectations between groups with different socio-demographic characteristics.

## Author contributions

All authors of this study meet the requirements for authorship and have approved the final version of the paper to be published. FR planned and conducted the data analysis, and drafted and revised the paper. CP contributed to the planning and data analysis, drafted and revised the paper. JR conceived of the study, provided oversight to its design and coordination, contributed to the interpretation of the data, and drafted and revised the paper. CM contributed to the design of the study, data collection tools, the interpretation of the data, and revised the paper. AM contributed to the design and coordination of the study and reviewed the paper. DB conceived of the study, contributed to its design and coordination, and revised the paper. DB is guarantor of the study.

## Conflicts of interest

None declared.

## Figures and Tables

**Table 1:** Descriptive statistics of socio-demographics, computer confidence and healthcare experience among the analysis sample of patients recruited from outpatient departments and GP surgeries in West London (*N* = 3157).

Variable	*N*	%
Age category		
18–24	291	9.2
25–34	902	28.6
35–44	719	22.8
45–54	492	15.6
55–64	346	11
65–74	263	8.3
75+	144	4.6

Sex		
Female	1908	60.4
Male	1249	39.6

Ethnicity		
White British	1739	55.1
White Non-British	658	20.8
British Black	235	7.4
Asian British	254	8
Other	271	8.6

Education		
None	131	4.1
GCSE	310	9.8
A-Levels	328	10.4
Vocational	366	11.6
Degree	1210	38.3
Higher degree	812	25.7

Confidence with using computers		
Not confident	274	8.7
Fairly confident	437	13.8
Confident	846	26.8
Very confident	1485	50.7

NHS services accessed in past 6 months		
0 to 2 types of services	2133	67.6
3+ types of services	1024	32.4

**Table 2:** Logistic regression model comparing those who did not expect to be asked for consent before access to their de-identified record (base = implicit consent model) and those who did expect to be asked for consent (explicit consent model), with respect to socio-demographic factors, computer confidence, healthcare experience and awareness of EHRs. *P*-values and 95% confidence intervals are adjusted for clustering by sampling site (*N* = 3157).

	Consent for de-identified record access (base: implicit consent)					
	Unadjusted OR		*P*	OR	Adjusted OR	
	OR	95% CI			95% CI	*P*
Sex (base: female)						
Male	0.66	(0.56,0.78)	<0.001	0.66	(0.56,0.78)	<0.001

Age (base: 25–34)						
18–24	1.21	(0.86,1.72)	0.27	1.07	(0.73,1.56)	0.75
35–44	1.07	(0.93,1.23)	0.33	1.05	(0.92,1.20)	0.45
45–54	1.13	(0.90,1.41)	0.29	1.15	(0.90,1.48)	0.26
55–64	0.88	(0.61,1.26)	0.49	0.83	(0.55,1.26)	0.39
65–74	1.09	(0.83,1.45)	0.52	0.94	(0.65,1.36)	0.75
75+	1.11	(0.83,1.49)	0.48	0.79	(0.53,1.17)	0.24

Ethnicity (base: White British)						
White Non-British	1.42	(1.18,1.71)	<0.001	1.46	(1.19,1.79)	<0.001
British Black	1.97	(1.45,2.66)	<0.001	1.71	(1.25,2.34)	<0.001
Asian British	1.67	(1.34,2.10)	<0.001	1.79	(1.37,2.34)	<0.001
Other	1.70	(1.40,2.06)	<0.001	1.66	(1.33,2.07)	<0.001

Education (base: higher degree)						
None	1.62	(1.07,2.47)	0.02	1.41	(0.89,2.23)	0.14
GCSE	1.68	(1.21,2.32)	<0.001	1.61	(1.09,2.36)	0.02
A level	1.54	(1.33,1.77)	<0.001	1.47	(1.26,1.72)	<0.001
Vocational	1.18	(0.92,1.51)	0.19	1.09	(0.82,1.44)	0.56
Degree	0.98	(0.84,1.15)	0.83	0.97	(0.86,1.10)	0.66

Computer confidence (base: very confident)						
Not confident	1.76	(1.47,2.09)	<0.001	1.65	(1.23,2.21)	<0.001
Fairly confident	1.43	(1.24,1.66)	<0.001	1.38	(1.12,1.70)	<0.001
Confident	1.07	(0.94,1.22)	0.31	1.05	(0.93,1.18)	0.47

NHS services accessed in the past 6 months (base: 0 to 2 types)						
3+ types	0.94	(0.82,1.08)	0.40	0.92	(0.81,1.05)	0.21

Awareness of EHRs (base: not aware)						
Aware of EHRs	0.67	(0.59,0.75)	<0.001	0.77	(0.67,0.88)	<0.001

**Table 3:** Logistic regression model comparing respondents who were aware of EHRs before the survey and respondents who were unaware (base = unaware), with respect to socio-demographics, computer confidence and healthcare experience. *P*-values and 95% confidence intervals are adjusted for clustering by sampling site (*N* = 3157).

	Aware of EHRs before the survey (base: unaware)					
	Unadjusted OR		*P*	OR	Adjusted OR	
	OR	95% CI			95% CI	*P*
Sex (base: female)						
Male	0.98	(0.83,1.16)	0.84	0.87	(0.75,1.01)	0.06

Age (base: 25–34)						
18–24	0.85	(0.59,1.25)	0.42	1.04	(0.68,1.60)	0.86
35–44	1.47	(1.23,1.77)	<0.001	1.76	(1.47,2.11)	<0.001
45–54	2.13	(1.69,2.68)	<0.001	2.87	(2.26,3.65)	<0.001
55–64	2.27	(1.80,2.86)	<0.001	3.91	(3.13,4.87)	<0.001
65–74	1.62	(1.34,1.95)	<0.001	3.59	(2.81,4.58)	<0.001
75+	0.96	(0.61,1.51)	0.86	3.06	(2.04,4.59)	<0.001

Ethnicity (base: White British)						
White Non-British	0.52	(0.42,0.66)	<0.001	0.43	(0.35,0.54)	<0.001
British Black	0.59	(0.43,0.81)	<0.001	0.64	(0.46,0.88)	0.01
Asian British	0.72	(0.56,0.92)	0.01	0.63	(0.49,0.82)	<0.001
Other	0.49	(0.39,0.62)	<0.001	0.48	(0.36,0.63)	<0.001

Education (base: higher degree)						
None	0.26	(0.16,0.43)	<0.001	0.23	(0.15,0.36)	<0.001
GCSE	0.41	(0.32,0.53)	<0.001	0.33	(0.28,0.39)	<0.001
A level	0.46	(0.33,0.65)	<0.001	0.45	(0.33,0.62)	<0.001
Vocational	0.51	(0.39,0.67)	<0.001	0.44	(0.35,0.56)	<0.001
Degree	0.72	(0.60,0.87)	<0.001	0.68	(0.58,0.81)	<0.001

Computer confidence (base: very confident)						
Not confident	0.34	(0.24,0.49)	<0.001	0.26	(0.18,0.38)	<0.001
Fairly confident	0.78	(0.66,0.92)	<0.001	0.64	(0.54,0.75)	<0.001
Confident	0.88	(0.75,1.03)	0.11	0.79	(0.67,0.93)	<0.001

NHS services accessed in the past 6 months (base: 0 to 2 types)						
3+ types	1.22	(0.90,1.70)	.02	1.27	(1.05,1.53)	0.01

**Table 4:** Univariable logistic regression analysis of missing data. *P*-values and 95% confidence intervals are adjusted for clustering by sampling site (Total *N* = 5331).

	Inclusion in complete sample (base: not included)		
	Missing	Unadjusted OR	
	*N* (%)	OR (95% CI)	*P*
*Age (base: 25–34)*	799 (15)		
18–24		1.13 (0.93,1.37)	0.20
35–44		0.87 (0.73,1.02)	0.09
45–54		0.66 (0.55,0.79)	<0.001
55–64		0.58 (0.48,0.70)	<0.001
65–74		0.40 (0.31,0.52)	<0.001
75+		0.27 (0.20,0.35)	<0.001
*Sex (base: female)*	611 (11.5)		
Male		1.00 (0.88,1.14)	0.98
*Ethnicity (base: White British)*	1109 (20.8)		
White Non-British		1.44 (1.15,1.79)	<0.001
British Black		0.64 (0.45,0.92)	0.02
Asian British		0.71 (0.49,1.02)	0.07
Other		0.94 (0.70,1.25)	0.66
*Education (base: higher degree)*	833 (15.6)		
None		0.30 (0.23,0.41)	<0.001
GCSE		0.52 (0.43,0.62)	<0.001
A level		0.68 (0.52,0.88)	<0.001
Vocational		0.70 (0.58,0.84)	<0.001
Degree		0.85 (0.71,1.02)	0.08
*Computer confidence (base: very confident)*	694 (13)		
Not confident		0.32 (0.25,0.42)	<0.001
Fairly confident		0.47 (0.37,0.61)	<0.001
Confident		0.81 (0.66,0.98)	0.03
*NHS services accessed in the past 6 months (base: 0 to 2 types)*	504 (9.5)		
3+ types		1.35 (1.14,1.59)	<0.001
*Awareness of EHRs (base: aware)*	276 (5.2)		
Previously unaware of EHRs		0.62 (0.56,0.68)	<0.001
*Consent expected for the use of de-identified record (base: implicit consent)*	952 (17.9)		
Expect to be asked for explicit consent		0.59 (0.52,0.65)	<0.001
*Consent expected for the use of identifiable record (base: implicit consent)*	695 (13)		
Expect to be asked for explicit consent		1.29 (1.04,1.60)	0.02
